# Simvastatin suppresses the DNA replication licensing factor MCM7 and inhibits the growth of tamoxifen-resistant breast cancer cells

**DOI:** 10.1038/srep41776

**Published:** 2017-02-02

**Authors:** Zheyong Liang, Wenjie Li, Jie Liu, Juan Li, Fang He, Yina Jiang, Lu Yang, Pingping Li, Bo Wang, Yaochun Wang, Yu Ren, Jin Yang, Zhijun Luo, Cyrus Vaziri, Peijun Liu

**Affiliations:** 1 Center for Translational Medicine, The First Affiliated Hospital of Xi’an Jiaotong University, Xi’an, Shaanxi 710061, P.R. China; 2 Key Laboratory for Tumor Precision Medicine of Shaanxi Province, The First Affiliated Hospital of Xi’an Jiaotong University, Xi’an, Shaanxi 710061, P.R. China; 3 Department of Pathology, The First Affiliated Hospital of Xi’an Jiaotong University, Xi’an, Shaanxi, 710061, P.R. China; 4 Department of Breast Surgery, The First Affiliated Hospital of Xi’an Jiaotong University, Xi’an, Shaanxi, 710061, P.R. China; 5 Department of Oncology, The First Affiliated Hospital of Xi’an Jiaotong University, Xi’an, Shaanxi, 710061, P.R. China; 6 Department of Biochemistry, Boston University School of Medicine, Boston, MA 02118, USA; 7 Department of Pathology and Laboratory Medicine, University of North Carolina, Chapel Hill, NC 27599, USA

## Abstract

Acquired tamoxifen resistance (TamR) remains a major challenge in breast cancer endocrine therapy. The mechanism of acquiring tamoxifen resistance remains elusive, and no effective drugs are available. In this investigation, we determined that the expression of the DNA damage marker γH2AX is upregulated under minichromosome maintenance protein 7 (MCM7) knockdown in phospho Ser807/811-retinoblastoma protein (p-Rb) defect cells. In addition, the expression of p-Rb was lower in TamR cells than in parental cells, and the expression of γH2AX was significantly upregulated when MCM7 was knocked down in TamR cells. Simvastatin, an agent for hypercholesterolemia treatment, activated the MCM7/p-RB/γH2AX axis and induced DNA damage in TamR cells, especially when combined with tamoxifen. Finally, *in vitro* and *in vivo* experiments demonstrated that simvastatin combined with tamoxifen increased TamR cell apoptosis and inhibited xenograft growth. In conclusion, simvastatin may suppress TamR cell growth by inhibiting MCM7 and Rb and subsequently inducing DNA damage.

Adjuvant endocrine therapies can halve the recurrence rate of estrogen receptor (ER)-positive breast cancer. However, approximately one in three ER-positive patients relapse during or after endocrine therapy^1^,^2^. Despite numerous studies of new markers and mediators of therapeutic resistance, effective drugs remain lacking^3^. Therefore, a better understanding of the molecular mechanisms underlying endocrine therapy resistance and the identification of targets that can overcome this resistance are urgently needed.

Tamoxifen, a selective estrogen receptor (ER) modulator, is most frequently used as an adjuvant endocrine therapy for women with ER-positive breast cancer^4^,^5^. Tamoxifen resistance in ER-positive breast cancer has been recently demonstrated to be associated with the activation of retinoblastoma protein (Rb). Recently, Bosco *et al*.^6^ reported that Rb-deficient cells partially bypass the cell-cycle blockade elicited by anti-estrogen therapy. Additionally, Lehn *et al*.^7^ showed that the functional status of Rb appears to be related to the patient’s response to tamoxifen treatment and that the loss of RB function is associated with approximately 40% of ER-negative cancer as opposed to approximately 5% of ER-positive cases. In fact, Rb deficiency is reported in approximately 20–35% of breast cancers^8^,^9^. Rb is a well-known G1/S checkpoint control protein^10^,^11^, but the association of Rb defect with the potential dysregulated DNA replication in tamoxifen-resistant breast cancer cells has not been established.

Minichromosome maintenance protein 7 (MCM7) is part of the MCM complex (MCM2–7) hexamer and a DNA replication licensing factor that plays a central role in eukaryotic DNA replication^12^,^13^. The MCM complex binds and translocates along the DNA, resulting in the unwinding of the DNA strand^14^. The suppression of MCMs increases the frequency of chromosome breaks and chromosome gaps in cells under replication stress^15^. Once the MCM complex is destroyed by downregulating any one of its subunits, cells will undergo limited replication and become hypersensitive to DNA replication stresses, easily causing DNA damage and further inhibiting cell growth via activating the checkpoint signals^15^. Therefore, we hypothesized that under the dysregulated DNA replication conditions caused by the Rb defect in tamoxifen-resistant cells, the additional destruction of the MCM complex may cause severe DNA damage.

We previously showed that simvastatin, an agent for the treatment of hypercholesterolemia^16^,^17^, reduces the expression of MCM7 and causes a significant upregulation of γH2AX expression; therefore, we proposed that simvastatin may cause DNA damage in tamoxifen-resistant breast cancer cells in which p-Rb expression is low via downregulation of MCM7.

In this study, we investigated the unconventional effects of the conventional drug simvastatin on breast cancer with tamoxifen resistance. We observed that simvastatin downregulated the expression of MCM7 and caused DNA damage in tamoxifen-resistant cells when combined with tamoxifen treatment. Notably, our results demonstrate for the first time that simvastatin, combined with tamoxifen, induces the apoptosis of tamoxifen-resistant cells and inhibits their growth both *in vitro* and *in vivo*.

## Results

### Characteristics of tamoxifen-resistant cells

Initially, we assessed the drug resistance of the TamR cell lines using MTT (Fig. S1A). Based on the results of the MTT assay, the IC50 in each cell line was as follows: MCF-7 4.378 μM; MCF-7 TamR 13.100 μM; T47D 5.120 μM and T47D TamR 11.146 μM. In addition, the tamoxifen resistance factor (RF) of each cell was calculated as follows: MCF-7 TamR 2.99 and T47D TamR 2.18. The TamR cells grew more slowly than the WT cells (Fig. S1B), and this difference was confirmed by cell-cycle analysis (Fig. S1C and D). Furthermore, we investigated markers of breast cancer stem cells in TamR and WT cells. Both MCF7 TamR and T47D TamR cells had a larger population of CD24-CD44 + cells and a smaller population of ALDH high-activity cells than the corresponding WT cells (Fig. S1E to H). These results indicate that our TamR cell line models share some characteristics of mesenchymal-like human breast cancer stem cells (CSCs)^18^.

### MCM7 knockdown increases γH2AX expression in tamoxifen-resistant breast cancer cells under an Rb-defective condition

**Figure 1:**
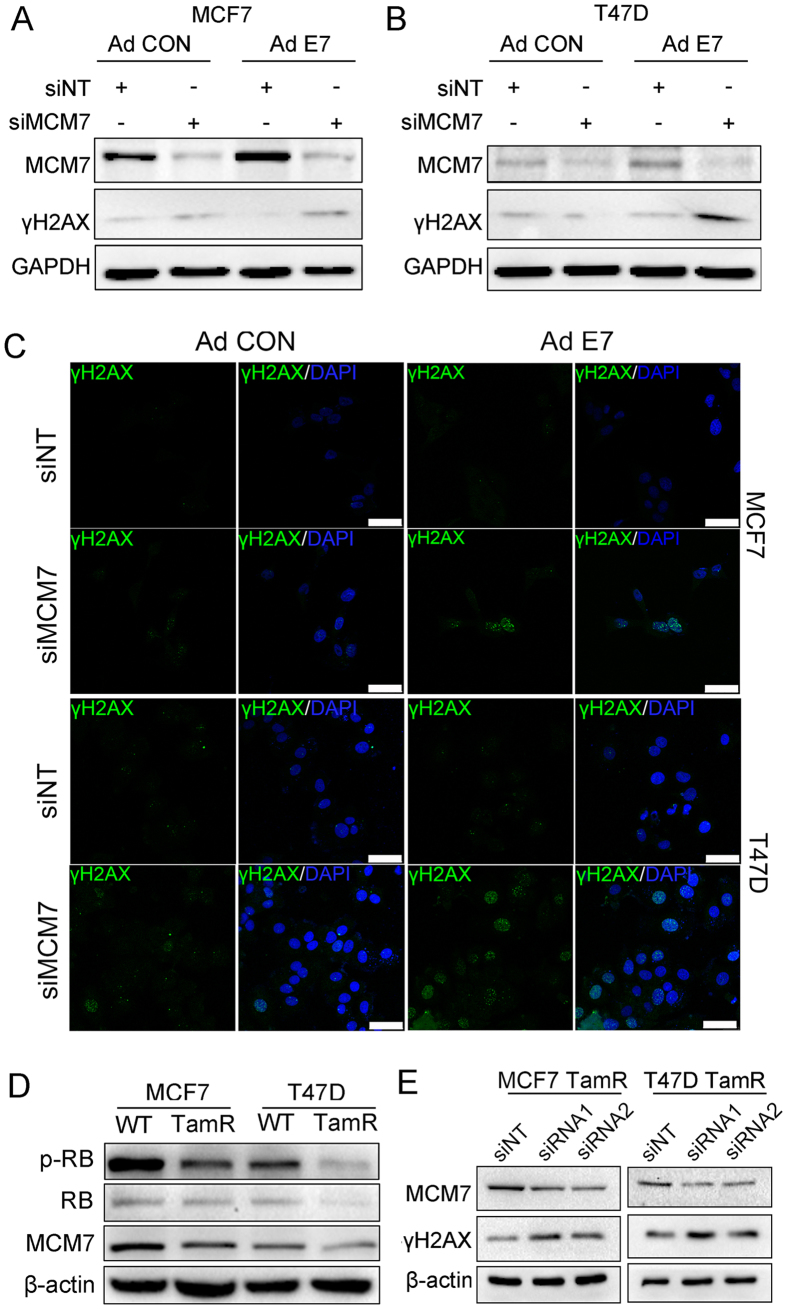
Downregulation of MCM7 induces γH2AX over-expression in tamoxifen-resistant breast cancer cells under the condition of an Rb defect. (**A**,**B**) Both MCF7 and T47D cells were infected with HPV E7 adenovirus (Ad E7) or control adenovirus (Ad CON) for 24 h and were then transfected with MCM7 siRNA or non-target siRNA (siNT). Forty-eight hours after transfection, the cells were lysed, and western blot was performed. (**C**) The MCF7 and T47D cells were infected with HPV E7 adenovirus (Ad E7) or control adenovirus vector (Ad CON) for 24 h and were then transfected with MCM7 siRNA. Forty-eight hours after transfection, the cells were fixed, and γH2AX was stained by immunofluorescence. The bars represent 50 μm. (**D**) The expression of p-RB, RB and MCF7 in wild-type cells and tamoxifen-resistant cells. (**E**) MCF7 TamR and T47D TamR cells were transfected with MCM7 siRNA or non-target siRNA (siNT). Forty-eight hours after transfection, the cells were lysed, and a western blot was performed.

We showed that MCM7 knockdown induces higher γH2AX expression in SaOS2 cells (Rb inactivated) than in U2OS cells (Rb permanently activated). Similar effects have been observed in other cell lines, such as SiHa (Rb inactivated) and C33A (Rb normally activated) (unpublished data). In this study, MCM7 was knocked down with two different siRNAs, and the Rb signal was inactivated by HPV E7 adenovirus (Ad E7)^19^. As expected, γH2AX was upregulated in the Rb/MCM7 double-defective MCF7 and T47D cells (Fig. 1A to C and Fig. S2A to C).

Recently, Lehn *et al*. demonstrated that Rb inactivity is an important characteristic of tamoxifen-resistant breast cancer in the clinic^7^. This finding inspired us to examine whether the Rb signal was also inactivated in our cell line models and whether MCM7 downregulation in TamR cells induces γH2AX upregulation. Western blot analysis showed that p-Rb expression was lower in MCF7 TamR and T47D TamR cells than that in their corresponding WT cells (Fig. 1D). Next, we knocked down MCM7 in MCF7 TamR cells and T47D TamR cells using two different siRNAs. Interestingly, γH2AX expression was also upregulated under MCM7 knockdown (Fig. 1E).

### Simvastatin combined with tamoxifen upregulates γH2AX and induces DNA damage in tamoxifen-resistant breast cancer cells

If downregulating MCM7 induces the upregulation of γH2AX in tamoxifen-resistant cells, drugs that cause MCM7 downregulation may induce DNA double-stranded breaks in tamoxifen-resistant cells. Previous studies by our group and other researchers^20^ have demonstrated that statins reduce the expression of MCM7; therefore, we investigated whether simvastatin also affects tamoxifen-resistant cells.

**Figure 2:**
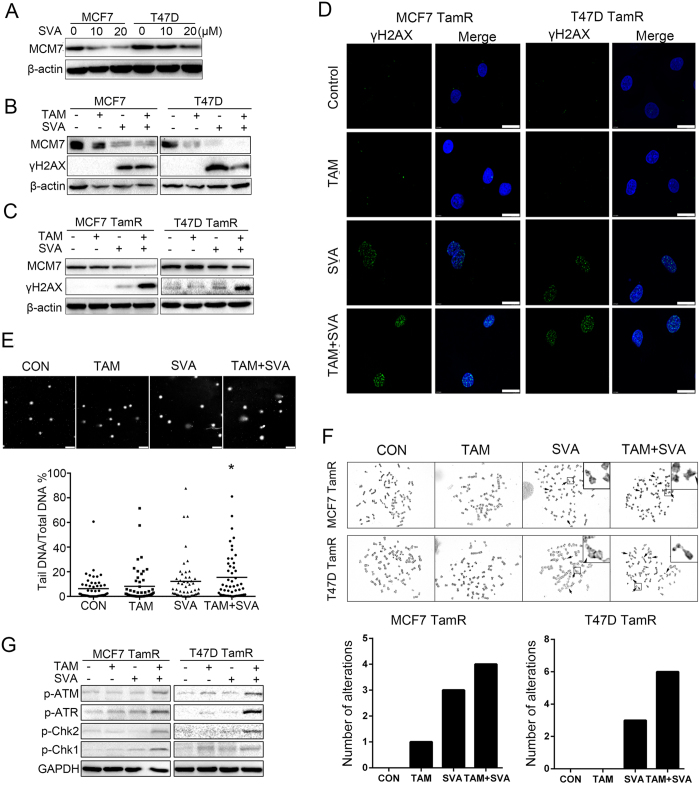
Simvastatin combined with tamoxifen upregulates γH2AX and induces DNA damage in tamoxifen-resistant breast cancer cells. (**A**) MCF7 and T47D cells were cultured at different doses (0 μM, 10 μM and 20 μM) of simvastatin. After treatment for 24 h, the cells were lysed, and a western blot was performed. (**B**) Four groups of MCF7 TamR and T47D TamR cells were cultured with a control solvent, 4-OH-tamoxifen (5 μM), simvastatin (20 μM), or 4-OH-tamoxifen (5 μM) plus simvastatin (20 μM). After treatment for 24 h, the cells were lysed, and a western blot was performed. (**C**) Four groups of MCF7 TamR and T47D TamR cells were cultured as described above. After treatment for 24 h, the cells were lysed, and a western blot was performed. (**D**) Four groups of MCF7 TamR and T47D TamR cells were cultured as described above. After treatment for 24 h, the cells were fixed, and immunofluorescence was performed. The images were obtained by fluorescence confocal microscopy; the bars represent 10 μm. (**E**) Four groups of MCF7 TamR cells were cultured as described above. After treatment for 24 h, comet assays were performed under alkaline conditions. Images were obtained by fluorescence confocal microscopy; the bar represents 100 μm. (**H**) Quantitative analysis of the DNA content in the comet tail relative to the total DNA content in each group of cells. Fifty cells were scored per group. *p < 0.05. (**F**) Four groups of MCF7 TamR and T47D TamR cells were cultured as described above. After treatment for 24 h, chromosome fragility analyses were performed, and representative images are shown. The chromosome gaps and breaks (indicated as arrows) were scored. Two metaphases were analyzed for each cell group. (**G**) Four groups of MCF7 TamR and T47D TamR cells were cultured as described above. After treatment for 24 h, the cells were lysed, and a western blot was performed.

We observed that the expression of MCM7 was downregulated after TamR cells were treated with simvastatin at a dose of 20 μM for 24 h (Fig. 2A). After treatment with either simvastatin (20 μM) alone or in combination with tamoxifen (5 μM) for 24 h, the expression of MCM7 was downregulated, and the expression of γH2AX was upregulated in wild-type MCF7 and T47D cells (Fig. 2B). Notably, western blot analysis showed that the expression of MCM7 was downregulated and that the expression of γH2AX was significantly increased in TamR cells after treatment with simvastatin combined with tamoxifen for 24 h (Fig. 2C). Immunofluorescence staining also showed that γH2AX foci formed in the nucleus after treatment with simvastatin combined with tamoxifen at both 24 h (Fig. 2D) and 72 h (Fig. S3A and B).

**Figure 3:**
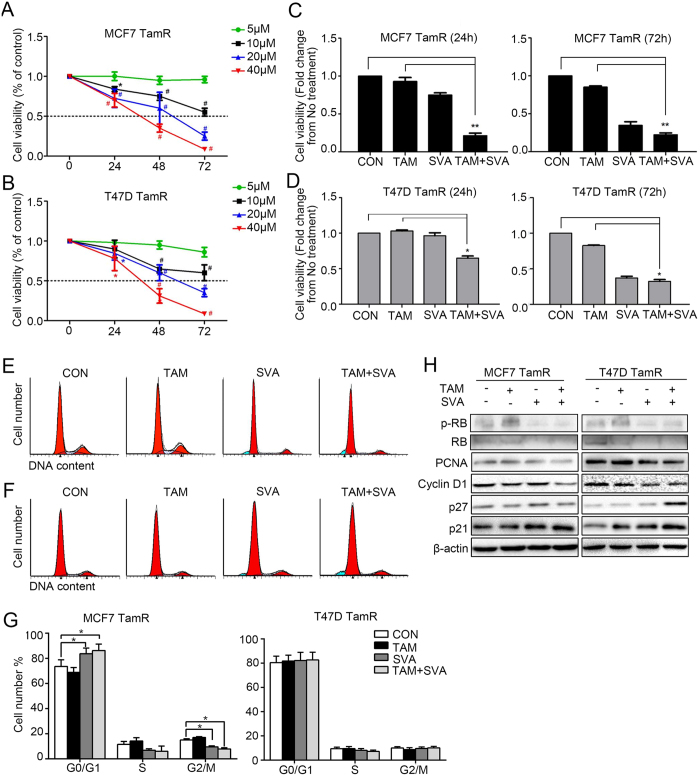
Simvastatin combined with tamoxifen inhibits the growth of tamoxifen-resistant breast cancer cells. (**A**,**B**) Both MCF7 TamR and T47D TamR cells were treated with simvastatin at doses ranging from 5 μM to 40 μM for 72 h; cell viability was measured by an MTT assay at 0 h, 24 h, 48 h and 72 h following treatment. (**C**,**D**) Four groups of MCF7 TamR or T47D TamR cells were cultured with solvent as a control, 4-OH-tamoxifen (5 μM), simvastatin (20 μM), or 4-OH-tamoxifen (5 μM) plus simvastatin (20 μM). After treatment for 24 h (**C**) or 72 h (**D**), cell viability was measured by an MTT assay. (**E**,**F**,**G**) Four groups of MCF7 TamR and T47D TamR cells were cultured as described above. After treatment for 24 h, the cell cycle was evaluated by flow cytometry in each group. (**H**) Four groups of MCF7 TamR and T47D TamR cells were cultured as described above. After treatment for 24 h, the cells were lysed, and a western blot was performed. n = 3, *p < 0.05, ^#^p < 0.01, **p < 0.01

Alkaline comet assays were performed to determine whether DNA damage occurred following treatment. DNA damage was more severe in MCF7 TamR cells than in control cells after treatment with simvastatin combined with tamoxifen for 24 h (Fig. 2E). We also estimated the extent of chromosomal damage after treatment in TamR cells by chromosome spread analyses. Colchicine was added to drive the cells into mitosis, and chromosome spreads were scored for signs of instability, such as breaks, gaps, and aberrant rearrangements (triradial or quadriradial chromosomes) (Fig. 2F). Furthermore, western blot analysis showed that the checkpoint proteins p-ATM, p-ATR, p-Chk1, and p-Chk2 were upregulated after treatment with simvastatin combined with tamoxifen for 24 h (Fig. 3G).

### Simvastatin combined with tamoxifen inhibits the growth of tamoxifen-resistant breast cancer cells

Because simvastatin induced DNA damage in TamR cells, we examined whether simvastatin inhibits the growth of TamR cells. Cell viability was assessed by MTT assay at 24 h, 48 h and 72 h after treatment with different concentrations of simvastatin. Simvastatin had little effect on either MCF7 TamR or T47D TamR cell growth at a low concentration (5 μM) but inhibited cell growth significantly at a high concentration (10 μM). In addition, the inhibitory effects of simvastatin were time- and concentration-dependent (Fig. 3A and B). Next, we observed that cell viability was inhibited when the cells were treated with simvastatin (20 μM) combined with tamoxifen (5 μM) for 24 h, and the growth of the cells was also inhibited after treatment with simvastatin alone for 72 h (Fig. 3C and D). Furthermore, cell-cycle analysis revealed that MCF7 TamR cells accumulated in G1 phase when treated with simvastatin alone or in combination with tamoxifen, but this accumulation was less notable in T47D TamR cells (Fig. 3E to G). Notably, western blot analysis indicated that the expression of p-Rb was downregulated and the expression of p27 was upregulated in TamR cells after treatment with simvastatin combined with tamoxifen for 24 h. However, the expression of PCNA, cyclin D1 and p21 did not exhibit significant changes (Fig. 3H).

### Simvastatin combined with tamoxifen induces apoptosis in tamoxifen-resistant breast cancer cells

**Figure 4:**
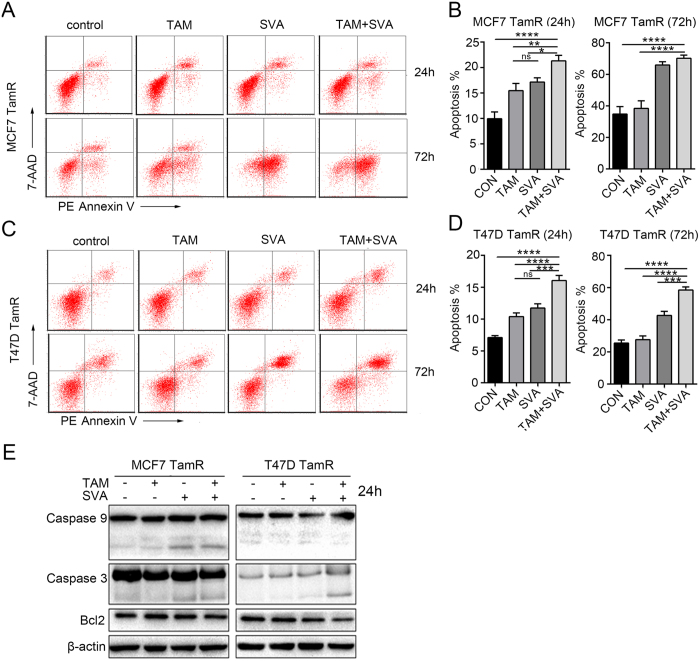
Simvastatin combined with tamoxifen induces apoptosis of tamoxifen-resistant breast cancer cells. (**A**,**B**,**C**,**D**) Four groups of MCF7 TamR and T47D TamR cells were cultured with solvent as a control, 4-OH-tamoxifen (5 μM), simvastatin (20 μM), or 4-OH-tamoxifen (5 μM) plus simvastatin (20 μM). After treatment for 24 h (**C**) or 72 h (**D**), the cells were digested and incubated with PE Annexin V and 7-AAD, and apoptotic cells were detected by flow cytometry. (**E**) Four groups of MCF7 TamR and T47D TamR cells were cultured as described above. After treatment for 24 h, the cells were lysed, and a western blot was performed. n = 3, *p < 0.05, **p < 0.01 and ****p < 0.0001.

Apoptosis assays were performed to investigate whether simvastatin induces apoptosis in tamoxifen-resistant cells. The number of apoptotic cells increased in MCF7 TamR or T47D TamR cells after treatment with simvastatin combined with tamoxifen for 24 h or 72 h (Fig. 4A to D). The number of apoptotic cells also increased in MCF7 TamR or T47D TamR cells after treatment with simvastatin alone for 72 h. As expected, western blot analysis revealed that cleaved caspase 3 and cleaved caspase 9 were upregulated in MCF7 TamR and T47D TamR cells after treatment with simvastatin combined with tamoxifen for 24 h, whereas Bcl2 exhibited little change following either treatment (Fig. 4E). We also observed that cleaved caspase 3 and cleaved caspase 9 were upregulated in MCF7 TamR cells after treatment with simvastatin alone for 72 h (Fig. S4). However, caspase, PARP and Bcl2 proteins were significantly degraded in T47D TamR cells after treatment with simvastatin alone or combined with tamoxifen for 72 h (Fig. S4). Thus, simvastatin combined with tamoxifen induces apoptosis in TamR cells.

### Simvastatin combined with tamoxifen inhibits the growth of tamoxifen-resistant breast cancer cells *in vivo*

**Figure 5:**
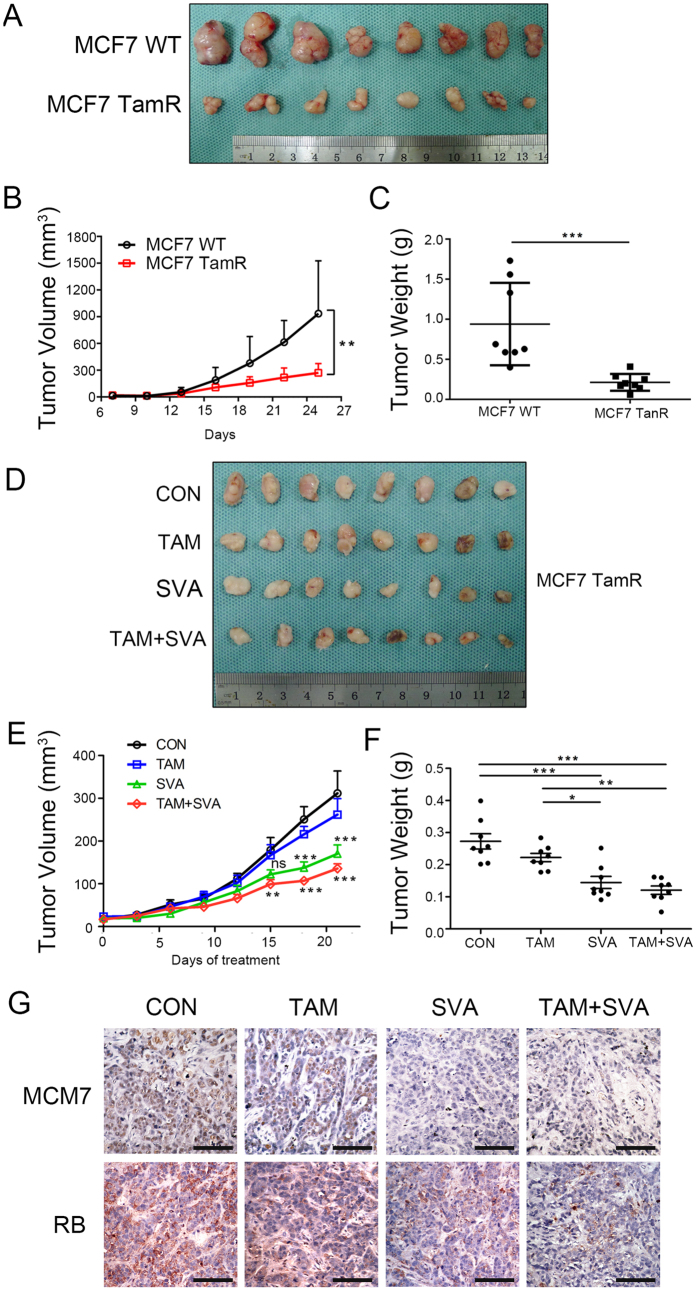
Simvastatin combined with tamoxifen inhibits the growth of tamoxifen-resistant breast cancer cells *in vivo*. Xenograft tumors were formed by injecting 2.5 × 10^6^ MCF7 or MCF7 TamR cells into the fat pads of SCID/Beige mice. (**A**) Tumors in the non-treated group are shown. (**B**,**C**) The xenograft tumors that formed from the MCF7 cells were significantly larger in size (**B**) and weight (**C**) than the tumors that formed from MCF7 TamR cells. (**D**,**E**,**F**) Six days after injection, the SCID/Beige mice were gavaged daily with placebo (CON) and tamoxifen (5 mg/kg) alone (TAM), simvastatin (30 mg/kg) alone (SVA) or tamoxifen (5 mg/kg) combined with simvastatin(30 mg/kg) (TAM + SVA) for 3 weeks. The xenograft size (**D**,**E**) and weight (**F**) are shown. (**G**) Immunochemical staining was performed to assess the expression of MCM7 and RB in the CON, TAM, SVA and TAM + SVA groups; the bars represent 75 μm. n = 8, *p < 0.05, **p < 0.01 and ***p < 0.001.

Xenograft tumor models were established in SCID/Beige mice using wild-type MCF7 cells and tamoxifen-resistant MCF7 cells to further investigate the *in vivo* effects of simvastatin. First, we assessed the tumorgenicity of these two cell lines. Approximately 2.5 × 10^6^ wild-type or tamoxifen-resistant MCF7 cells were injected into the fat pads of six-week-old SCID/Beige mice. Consistent with the findings of the *in vitro* experiment, the tumors formed by MCF7 TamR cells grew more slowly than those formed by wild-type MCF7 cells (Fig. 5A to C). Next, seven days after the injection, when the xenograft tumors were palpable, the mice injected with MCF7 TamR cells were randomly allocated to either tamoxifen (5 mg/kg) alone, simvastatin (30 mg/kg) alone or tamoxifen (5 mg/kg) combined with simvastatin (30 mg/kg) by gavage daily. The tumor volumes were measured every 3 days. After three weeks, the tumor size and weight decreased remarkably in the mice treated with simvastatin combined with tamoxifen compared with the mice in the placebo group (Fig. 5D to F). Furthermore, immunochemistry staining revealed lower MCM7 expression in the xenograft tumors in the simvastatin combined with tamoxifen group (Fig. 5G). Taken together, these data support the hypothesis that simvastatin suppresses TamR cell growth and inhibits MCM7 expression.

## Discussion

In the present study, we initially established two tamoxifen-resistant cell lines and tested the effects of simvastatin on these cells. Our data indicated that simvastatin upregulated γH2AX and induced DNA damage in tamoxifen-resistant cells. In addition, simvastatin inhibited the growth of tamoxifen-resistant cells and induced apoptosis in these cells when administered in combination with tamoxifen. Importantly, the expression of MCM7 and Rb was downregulated under simvastatin treatment; this downregulation may be the mechanism underlying the growth-inhibiting effects of simvastatin. The growth-inhibiting effect of simvastatin on tamoxifen-resistant cells was also confirmed in our *in vivo* studies. Taken together, these results suggest that simvastatin may be a potential treatment for tamoxifen-resistant breast cancer patients.

Statins are competitive inhibitors of 3-hydroxy-3-methylglutaryl-coenzyme A (HMG-CoA) reductase, a rate-limiting enzyme that converts HMG-CoA to mevalonate in the synthesis of cholesterol^16^,^17^. In addition to their original role in lowering serum cholesterol levels, accumulating evidence suggests that statins may inhibit carcinogenesis^21^,^22^,^23^,^24^,^25^,^26^,^27^ and that the anticancer effect of statins can be potentially exploited for cancer therapy^28^,^29^. Retrospective studies have concluded that the long-term use of statins reduces the risk of colorectal cancers^30^. However, the anti-tumor targets of simvastatin remain elusive. In our study, we investigated the effects of simvastatin on tamoxifen-resistant breast cancer cells and determined that MCM7 downregulation may contribute to simvastatin’s effects.

The MCM complex, as an important DNA replication initiation factor^12^, is a key regulator of the cell cycle. The MCM complex participates in the formation of the pre-replication complex, which assembles at replication origins during the early G1 phase^31^,^32^,^33^,^34^ and is responsible for the correct licensing of DNA. Ibarra and his colleges^15^ demonstrated that knockdown any one of the MCM complex subunits (MCM2-7) will lead to dysfunction of the whole complex and reduce the backup capacity of DNA licensing, which then leads to abnormal replication of DNA during S phase and activates the DNA damage response (DDR) to stop the cell cycle. In fact, downregulating MCM7 alone also activates DDR by regulating Rad17^35^,^36^. Our data showed that simvastatin downregulated MCM7 in TamR cells, which in turn induced the upregulation of γH2AX. These observations imply that MCM7 contributes to the growth-inhibiting effects of simvastatin. MCM7 may not be the only target of simvastatin. Archana Gopalan *et al*.^37^ found the combination of that simvastatin with γ-tocotrienols reduced the number of stem-like cells in tamoxifen-resistant breast cancer cells. We observed that simvastatin also reduced the RB signals and influenced the expression of cyclinD1 and p27 in TamR cells. Our *in vivo* experiment shown that simvastatin alone reduced the growth of tumor significantly but the effect of tamoxifen combined with simvastatin does not look different from the effect of simvastatin alone. It indicated that simvastatin may didn’t restore the tamoxifen sensitivity of the cells *in vivo*. Other mechanism that independent of the hormone receptor pathway may contribute to the tumor growth inhibition effects of simvastatin. Based on our results, we assume that under the uncontrolled cell-cycle progression caused by the Rb defect in TamR cells, the additional inactivation of the MCM complex reduces the backup capacity of DNA licensing, which then causes lethal DNA damage and further contributes to apoptosis in tamoxifen-resistant cells. In fact, an Rb signal defect is observed in approximately 20–35% of breast cancers^8^,^9^, and thus whether simvastatin also inhibits other Rb-defect tumor cells merits further investigation.

In summary, we established two tamoxifen-resistant cell lines and determined that downregulating MCM7 in TamR cells induced the upregulation of γH2AX. We also observed that simvastatin downregulated MCM7 and induced DNA damage in TamR breast cancer cells. Furthermore, simvastatin inhibited the growth and induced apoptosis in TamR cells. Our data indicate that the growth-inhibitory effects of simvastatin are probably achieved by downregulating MCM7 when defective Rb signals are present. This study provides support for the further evaluation of simvastatin as a new strategy for the treatment of patients with tamoxifen-resistant breast cancer.

## Methods

### Cell culture and treatments

Human MCF7 and T47D breast carcinoma cells, which were purchased from Shanghai Institute of Biochemistry and Cell Biology, Chinese Academy of Sciences (Shanghai, China), were routinely grown in Dulbecco’s modified Eagle’s medium (DMEM; Hyclone, USA) containing 5% fetal bovine serum (FBS; Gibco, USA). The cell lines were authenticated by a short-tandem repeat analysis by the cell bank. The tamoxifen-resistant sublines (MCF7 TamR or T47D TamR) were derived from MCF-7 or T47D by continuous exposure to tamoxifen as previously described^38^. In brief, a single-cell suspension of MCF7 or T47D cells was incubated at a high density and then cultured for 30 days in DMEM (5% FCS) supplemented with 1 μM 4-hydroxytamoxifen (Sigma-Aldrich, USA). Massive cell death occurred after 5–10 days of drug exposure, and colonies emerged after 20 days of drug exposure. The developed colonies (>3 mm diameter) were examined separately, and each colony was isolated and transferred to a well of a 96-well cell-culture plate. After several rounds of drug selection, the TamR monoclonal sublines were established and maintained in phenol-free DMEM containing 5% charcoal-dextran stripped FBS supplemented with 4-hydroxytamoxifen (1 μM in ethanol). Before each experiment, the cells were transferred to phenol-free DMEM containing 5% charcoal-dextran stripped FBS, except where noted.

### Cell viability assay

The cells were seeded in 96-well plates at 5 × 10^3^ cells/well. On day 2, the cells were treated with different concentrations of TAM or SVA for the time period indicated. Vehicle (0.1% ethanol or DMSO) was used as a control. At the end of the treatment, 20 μl of 5 mg/ml MTT was added to the medium and incubated for 4 hours at 37 °C. After removing the medium, 150 μl of MTT solvent (DMSO) was added to each well for 15 minutes, and the optical density (OD) values were read using a microplate reader (PerkinElmer, Waltham, MA, USA) (λ = 490 nm). Each experiment was repeated three times. All OD values were normalized by converting them to a percentage of the mean control value.

### Analysis of the cell cycle and apoptosis

After incubation, the cells were harvested, fixed in cold 70% ethanol, and stored at −20 °C for at least 20 min. After a PBS wash, the cell pellets were resuspended in 50 g/ml propidium iodide (PI) containing 20 g/ml RNase for 30 min. Then, the cell DNA content was measured by propidium iodide staining and flow cytometry (FACSort, Becton-Dickinson) using the Cell Quest software program. At least 10,000 cells were counted in each sample. The forward and side scatters were gated to exclude contributions from cell debris. Cell apoptosis was assayed using a PE Annexin V Apoptosis Detection Kit (BD Biosciences; USA).

### siRNA and adenovirus

One hundred picomoles of siRNA and 5 μl of Lipofectamine™ 2000 (Invitrogen, Life Technologies, Shanghai, China) were each diluted separately in 245 μl of opti-MEM medium (Gibco), respectively. The diluted siRNA and Lipofectamine™ 2000 reagents were mixed and incubated at room temperature (RT) for 15 minutes. The siRNA-lipid complex was added to cells that had been grown to 70–80% confluence in six-well plates, and the cells were incubated at 37 °C in a 5% CO_2_ incubator. The sequences of the sense and antisense primers were as follows: MCM7 siRNA#1 sense: 5′-AUCGGAUUGUGAAGAUGAATT-3′; antisense: 5′-UUCAUCUUCACAAUCCGAUTT-3′ and MCM7 siRNA#2 sense: 5′-GCUCCAGAUUCAUCAAAUUTT-3′; antisense: 5′-AAUUUGAUGAAUCUGGAGCTT-3′ and negative control (NC) siRNA sense: 5′-UAGCGACUAAACACAUCAATT-3′; antisense: 5′-UUGAUGUGUUUAGUCGCUATT-3′. The HPV E7 adenovirus and an ‘empty’ control adenovirus vector were packaged, and the cells were infected as previous described^19^.

### Western blot assay

The cells were lysed with RIPA buffer supplemented with protease inhibitors (Roche, NJ, USA) and phosphatase inhibitors. The protein lysates were separated by 10% SDS-PAGE and transferred to PVDF membranes (Bio-Rad, CA, USA), and the membranes were incubated with the indicated antibodies. The PARP, caspase-3, caspase-9, PCNA, γH2AX, p-RB, p-ATM, p-ATR, p-Chk1 and p-Chk2 primary antibodies were obtained from Cell Signaling Technology, USA. The MCM7, p21, p27 and Bcl2 primary antibodies were obtained from Santa Cruz, USA. The Rb, cyclin D1, GADPH and β-actin primary antibodies were obtained from Proteintech, China.

### Immunofluorescence

The cells were cultured on chamber slides in DMEM and were fixed with a 4% paraformaldehyde solution for 15 min at RT after being treated for 24 h or 72 h. The cells were washed three times with PBS and then permeabilized with 0.2% Triton X-100 for 10 min. The slides were blocked with 5% BSA and 10% horse serum in PBST for 1 h at RT and were then incubated with antibodies against γH2AX (1:200) (Cell Signal Technology, #9718P; USA) at 4 °C overnight. After rinsing with PBST three times, the cells were incubated with an Alexa Fluor 488 secondary antibody (Invitrogen #A11008; USA) (1:200) for 45 min at RT. Then, the cells were washed twice and were stained with 5 μg/ml DAPI, followed by imaging using a confocal microscope (Leica SP5II).

### Comet Assay

Alkaline comet assays were performed on MCF7 TamR cells using a Single Cell Gel Electrophoresis Assay Kit (Trevigen, Inc., USA) according to the manufacturer’s instructions. One hundred cells were spotted onto each sample area, and 50 cells from each group were analyzed and quantified using the CASP1.2.3 beta1 software (Krzysztof Konca, Comet Assay Software Project Lab, http://caspla.com).

### Chromosome spread analyses

The cells were treated with 0.5 g/ml of colchicine for 5 h at 37 °C before collection. To prepare the metaphase spreads, the cells were resuspended in 75 mM KCl, incubated for 15 min at 37 °C, centrifuged, and resuspended in fixation solution (3:1 vol/vol methanol/acetic acid). One hundred microliters of the cell suspension was dropped onto pre-cleaned microscope slides and dried overnight. The metaphase chromosomes were visualized by Giemsa staining.

### Animals, xenotransplantation and treatments

All animal experiment protocols were approved by the Institutional Animal Care and Use Committee of the First Affiliated Hospital of Xi’an Jiaotong University. The methods were conducted in accordance with the approved guidelines. A total of 2.5 × 10^6^ cells resuspended in 200 ml of PBS was injected subcutaneously into the fat pads of six-week-old female SCID/Beige mice (Centre of Laboratory Animals, The Medical College of Xi’an Jiaotong University, Xi’an, China). Tamoxifen (5 mg/kg in peanut oil) was administered daily by gavage as previously described^39^. Simvastatin (30 mg/kg in distilled water) was administered daily by gavage. Tumor volume was calculated using the following formula: (long axis × short axis^2^)/2.

### Immunohistochemical staining

Immunohistochemistry was performed on paraformaldehyde-fixed paraffin sections. The paraffin-embedded samples were cut into 4-μm-thick sections, which were baked at 60 °C for at least 6 hours. The paraffin sections were then deparaffinized in xylene and rehydrated in graded ethanol. Antigen retrieval was performed by treatment with sodium citrate buffer for 2 minutes in a pressure cooker. After antigen retrieval, endogenous peroxidase activity was quenched in 3% hydrogen peroxide for 10 min, followed by blocking with goat plasma at 37 °C for 30 minutes and incubation with the MCM7 antibody (1:100, Santa Cruz; USA) or Rb antibody (1:100, Proteintech; China) at 4 °C overnight. A biotinylated secondary antibody (ZSGB-Bio, Beijing, China) was used to detect the primary antibody. Then, the sections were incubated with diaminobenzidine before counterstaining with hematoxylin. Finally, the sections were dehydrated in graded ethanol and transparentized in xylene.

### Statistical analysis

Statistical analysis was performed using GraphPad Prism version 6.00 for Windows (GraphPad Software, La Jolla California USA, www.graphpad). The values are expressed as the mean ± SD. Comparisons between two groups were performed using an unpaired Student’s t-test. Two-way ANOVA followed by Dunnett’s multiple comparisons test was used for multiple comparisons. All statistical tests were two-sided. p < 0.05 was considered an indicator of statistical significance.

## Additional Information

**How to cite this article**: Liang, Z. *et al*. Simvastatin suppresses the DNA replication licensing factor MCM7 and inhibits the growth of tamoxifen-resistant breast cancer cells. *Sci. Rep.* **7**, 41776; doi: 10.1038/srep41776 (2017).

**Publisher's note:** Springer Nature remains neutral with regard to jurisdictional claims in published maps and institutional affiliations.

## Supplementary Material

Supplementary Information
