# Supplementary material for: Simvastatin suppresses the DNA replication licensing factor MCM7 and inhibits the growth of tamoxifen-resistant breast cancer cells
Source: Sci Rep. 2017 Feb 2;7:41776. doi: 10.1038/srep41776 (PMC5288718; doi:10.1038/srep41776)
Supplement: Supplementary Information [file srep41776-s1.pdf]

Simvastatin suppresses the DNA replication licensing factor MCM7 and inhibits the growth of tamoxifen-resistant breast cancer cells

Zheyong Liang[1,2]*, Wenjie Li[1,2]*, JieLiu[1,2], Juan Li[1,2], Fang He1, Yina Jiang[3], Lu Yang[1], Pingping Li[1,2], Bo Wang[1,2], Yaochun Wang[1,2], Yu Ren[4], Jin Yang[5], Zhijun Luo[6], CyrusVaziri[7], Peijun Liu[1,2]

*These authors contributed equally to this work and should be considered co-first authors

Author information:

1. Center for Translational Medicine, The First Affiliated Hospital of Xi’an Jiaotong University, Xi’an, Shaanxi 710061, P.R. China

2. Key Laboratory for Tumor Precision Medicine of Shaanxi Province, The First Affiliated Hospital ofXi’an Jiaotong University, Xi’an, Shaanxi 710061, P.R. China

3. Department of Pathology, The First Affiliated Hospital of Xi’an Jiaotong University, Xi’an, Shaanxi, 710061, P.R. China

4. Department of Breast Surgery, The First Affiliated Hospital of Xi’anJiaotong University, Xi’an, Shaanxi, 710061, P.R. China

5. Department of Oncology, The First Affiliated Hospital of Xi’an Jiaotong University, Xi’an, Shaanxi, 710061, P.R. China

6. Department of Biochemistry, Boston University School of Medicine, Boston, MA 02118, USA

7. Department of Pathology and Laboratory Medicine, University of North Carolina, Chapel Hill, NC 27599, USA

Supplemental Figure 1:

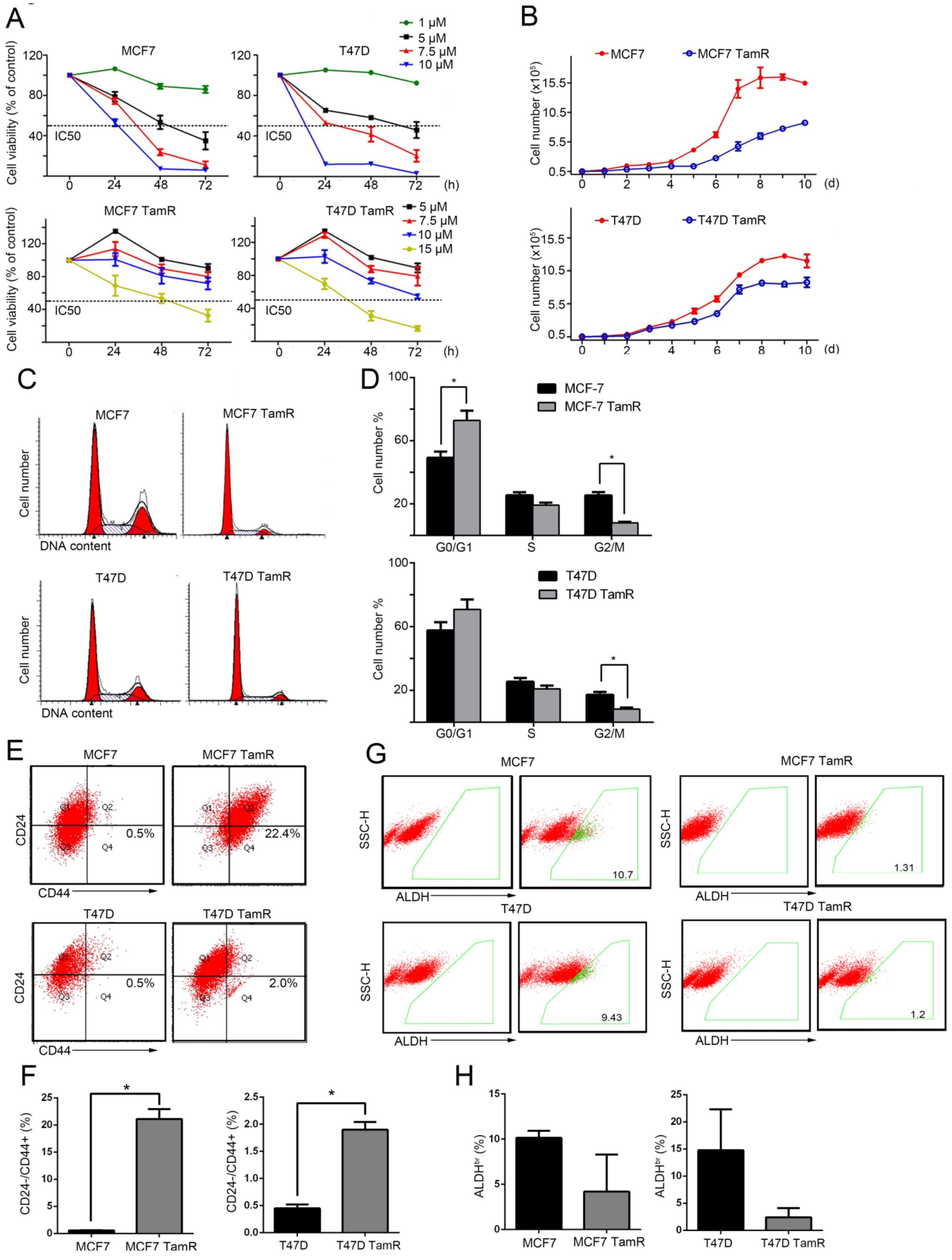

Supplemental Figure 1:

(A) Both wild-type and tamoxifen-resistant MCF7 or T47D cells were treated with 4-OH-tamoxifen at doses ranging from 1 μM to 15 μM for 72 h, and cell viability was measured by an MTT assay at 0 h, 24 h, 48 h and 72 h following treatment. The percentage of cell viability was calculated from the OD values of the test groups normalized to the control groups. (B) Wild-type and tamoxifen-resistant cells were cultured under the same conditions, and the cell numbers were counted daily for 10 days. (C, D) After culturing for 48 h, both the wild-type and tamoxifen-resistant cells were collected, and the cell cycles were analyzed by flow cytometry. (E, F) Wildtype and tamoxifen resistant cells were collected and incubated with CD24 and CD44 specific antibodies, then CD24-/CD44+ cells were counted by flow cytometry. (G, H) Wildtype and tamoxifen resistant cells were collected and incubated with ALDH specific antibody with or without ALDH inhibitor, then cells were tested by flow cytometry and ALDHbright cells were selected by being compared with the ALDH inhibitor control groups. n=3, * $p<0.05$

Supplemental Figure 2:

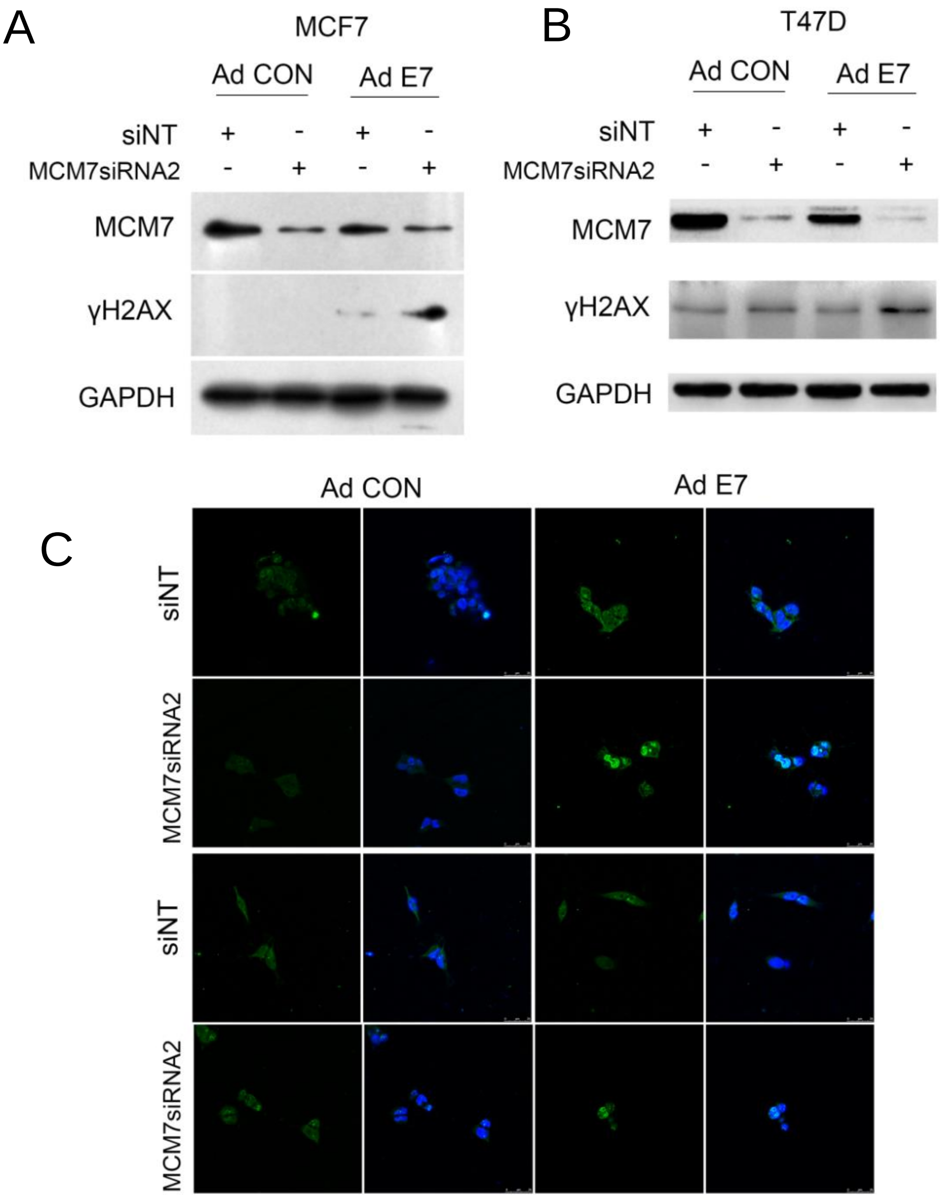

Supplemental Figure 2:

(A, B) Both MCF7 and T47D cells were infected with HPV E7 adenovirus (Ad E7) or control adenovirus (Ad CON) for 24 h and were then transfected with MCM7 siRNA or non-target siRNA (siNT). Forty-eight hours after transfection, the cells were lysed, and western blot was performed. (C) The MCF7 and T47D cells were infected with HPV E7 adenovirus (Ad E7) or control adenovirus vector (Ad CON) for 24 h and were then transfected with MCM7 siRNA. Forty-eight hours after transfection, the cells were fixed, and γH2AXwas stained by immunofluorescence. The bars represent 25 μm.

Supplemental Figure 3:

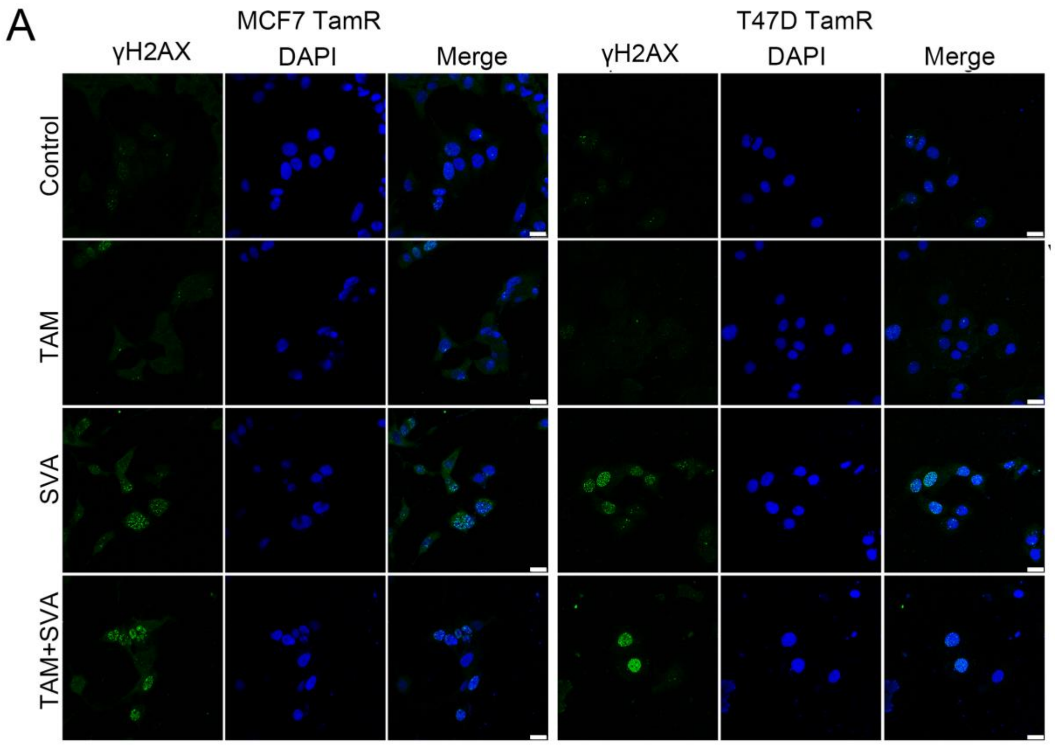

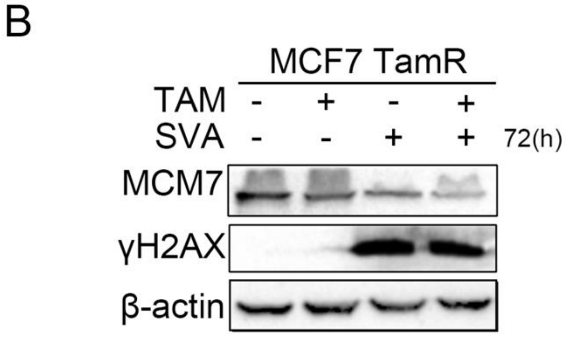

Supplemental Figure 3:

(A) Four groups of MCF7 TamR and T47D TamR cells were cultured with control solvent,4-OH-tamoxifen (5μM),simvastatin (20μM)and4-OH-tamoxifen (5μM) plussimvastatin (20μM),respectively. After being treated for 72hs, cells were fixed and immunofluorescence wasdone. The images were taken by fluorescence confocal microscopy;barsstand for 25μm. (B) Four groups of MCF7 TamRand T47D TamR cells were cultured as above mentioned.Afterbeingtreated for 72hs,cells were lysed and western blot were done.

Supplemental Figure 4:

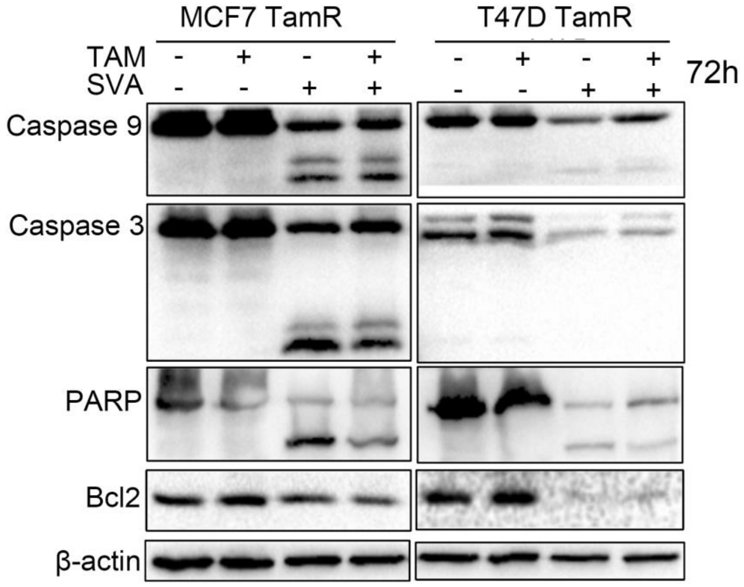

Supplemental Figure 4:

Four groups of MCF7 TamR and T47D TamRcells were cultured with solvent as a control, 4-OH-tamoxifen (5 μM),simvastatin (20 μM), or 4-OH-tamoxifen (5 μM) plus simvastatin (20 μM).After treatment for 72h, the cells were lysed, and a western blot was performed.
